# Illuminating the genomic frontier of invasive non‐typhoidal *Salmonella* infections

**DOI:** 10.1002/ctm2.70526

**Published:** 2025-11-12

**Authors:** Hao Wang, Li Tang, Haiyang Zhou, Peilu Xie, Min Yue

**Affiliations:** ^1^ School of Life Science Hangzhou Institute for Advanced Study University of Chinese Academy of Sciences Hangzhou China; ^2^ Department of Veterinary Medicine Zhejiang University College of Animal Sciences Hangzhou China; ^3^ State Key Laboratory for Diagnosis and Treatment of Infectious Diseases National Clinical Research Center for Infectious Diseases National Medical Center for Infectious Diseases The First Affiliated Hospital College of Medicine Zhejiang University Hangzhou China

In October 2024, the World Health Organisation (WHO) designated non‐typhoidal serovars of *Salmonella enterica* as a global high‐risk agent for Public Health Emergency of International Concern (PHEIC), spotlighting this urgent threat to global public health.^1^ Unlike non‐invasive serovars, which typically cause gastroenteritis, the invasive non‐typhoidal *Salmonella* (iNTS) ones drive severe extraintestinal infections, responsible for approximately 87,100 deaths annually, with mortality rates of 18.1%.^3^ The burden is critically compounded by the escalating crisis of antimicrobial resistance (AMR).

While historically recognised as a major public health issue in Africa,^4^ often associated with HIV and malaria co‐infections,^5^ its epidemiology and transmission dynamics in other regions remain poorly understood. To fill the gap, our group has reported several invasive non‐typhodial *Salmonella* serovars (Goldcoast, Livingstone, Telelkebir and Uzaramo) circulating.^6^, ^7^, ^8^, ^9^ Most recently, our large‐scale genomic epidemiology study in China,^10^ combining whole‐genome sequencing with advanced Bayesian analyses, has uncovered a disturbing evolutionary shift from serovar Choleraesuis to Enteritidis. Challenging the conventional understanding of iNTS as a zoonotic disease transmitted from animals, our genomic evidence, as well as the patient cohort, demonstrates that iNTS is adapting to humans and evolving toward sustained human‐to‐human transmission.^10^ Growing recognition of the bacterium's pandemic potential demands an urgent revision of global surveillance and targeted interventions.

## 1 SHIFTING SEROVAR AND RISING AMR

Analysing the whole‐genome sequencing (WGS) data of iNTS collected over the past three decades in China, the recent study revealed a significant epidemiological shift in China: The predominant serovar has transitioned from *S*. Choleraesuis, traditionally associated with swine, to *S*. Enteritidis,^10^ a serovar notorious for its global outbreak‐prone and frequent association with poultry.^11^, ^12^, ^13^ This change indicates possible adaptive evolution driven by environmental changes (e.g. surge of poultry consumption, targeted interventions) or host interactions (e.g. immune pressure). Alarmingly, the genomic analysis^10^ highlights a surge of AMR—86.54% of the iNTS strains possessing quinolone resistance, either through genetic mutations (e.g. *gyrA* mutations) or acquired genes (e.g. *qnr* genes). Furthermore, 66% of the isolates were multidrug‐resistant (MDR). Of particular concern is the annually increasing detection rate of *bla_CTX‐M_* genes, conferring resistance to third‐generation cephalosporins. Genetic context and co‐localisation analyses implicate mobile genetic elements (MGEs)—plasmids, transposons, and integrons—as key drivers of resistance gene dissemination. The MGEs carrying resistance determinants facilitate horizontal gene transfer across bacterial populations, challenging the efficacy of frontline therapeutics like fluoroquinolones and cephalosporins. To counter the global trend of rising resistance in Enterobacteriaceae, the heightened surveillance of MGE dynamics and targeted interventions to disrupt the dissemination of MGE are urgently needed.

## 2 AGE AND GENDER DISPARITIES

Integrating strain‐specific genomic data with national population statistics, a novel risk index model assessed the susceptibility to iNTS infections across different age and gender groups in China.^10^ The finding demonstrated that infants younger than one year are at a disproportionately high risk, with infection rates over 15 times greater than those in other age groups.^10^ This demographic's heightened susceptibility is likely attributable to a combination of factors, including immature immune systems and exposure risks like contaminated human food or close contact with the pathogen carriers. Moreover, the study reveals a significant gender disparity, with males experiencing a significantly higher incidence of iNTS infections compared to females.^10^ This gender‐based difference mirrors patterns observed in various other bacterial infections, suggesting potential biological (e.g. hormonal influences on immune response) or behavioural factors (e.g., occupational exposure) that warrant further investigation^15^. These insights underscore the urgent need for targeted public health interventions, such as enhanced neonatal screening and gender‐specific risk mitigation strategies, to curb iNTS morbidity and mortality.

## 3 GLOBAL EVOLUTION AND LOCAL ADAPTATION

By employing advanced Bayesian spatiotemporal models with comprehensive global phylogenomic analyses, we resolved the *S*. Enteritidis Global Epidemic Clade previously defined^4^ into three distinct subclades: Global‐a (emerged in 1965–1975), Global‐b (1970–1981) and Global‐c (2000–2004).^10^ This finding provides a refined framework for understanding the global epidemiology and evolutionary trajectory. Notably, the distribution of Global‐b in South Africa and China suggests potential intercontinental dissemination of the pathogen, likely facilitated by international travel or trade routes, such as the exportation of poultry breeding stocks from Europe to Asia.^12^ The Global‐c, primarily localised to China, is distinguished by its elevated resistance profiles (MDR rate: Global‐c, 97.56% > Global‐b, 22.36% > Global‐a, 2.07%).^10^ Pan‐genome analysis further reveals that this clade has accumulated distinct mutations in genes governing a range of core biological functions—including metabolism, information processing, and cellular signalling—differentiating it from African clades like the C&E lineages.^10^ The genetic divergences reflect a complex evolutionary history driven by global dissemination and region‐specific adaptation, which establishes the necessity of clade‐specific genomic surveillance to monitor the spread and adaptation of iNTS.

## 4 GENOMIC EVIDENCE OF HUMAN‐TO‐HUMAN TRANSMISSION

The paradigm‐shifting finding in the study is the predominance of human‐to‐human transmission in the spread of iNTS.^10^ By combining base substitution rates with spatiotemporal correlation analyses, we established that pairs of isolates with single‐nucleotide polymorphism (SNP) distances of four or fewer are indicative of recent transmission events. Through the analysis of more than 800 *S*. Enteritidis genomes sourced from a One Health perspective, encompassing animals, invasive human cases, and diarrheal patients, the study determined that 57.52% of transmission events leading to invasive disease are attributable to direct human‐to‐human contact.^10^ Notably, diarrheal patients are posited as key intermediaries in this transmission chain, facilitating the spread of the pathogen within human populations, potentially through close contact or humanprepared‐food contamination. This conclusion is bolstered by significant genomic differences between human‐ and animal‐derived strains, as evidenced by MGE distribution, resistance gene profiles, and pan‐genome data, shifting the conventional livestock‐centric transmission model and aligning with preliminary evidence from our group.^14^ The finding emphasises an urgent need for revised public health strategies focusing on human contact networks.^10^ Using a ≤4 SNP threshold is recommended in genomic analyses to trace outbreaks and enable early interventions^10^.

## 5 STRATEGIC INTERVENTIONS AND FUTURE DIRECTIONS

To date, no vaccine has achieved regulatory approval for human use. To combat the global emergence of iNTS with increasing human‐adapted transmission, we propose key strategies including: (1) Mandating iNTS in routine surveillance of bloodstream infection, particularly in hotspot areas; (2) Implementing strict antimicrobial stewardship programs in hospitals and clinics, particularly avoiding empirical use of fluoroquinolones or 3rd‐gen cephalosporins unless indicated; (3) Expanding One Health epidemiological investigations combining human case data, animal surveillance, food chain sampling, and environmental testing using WGS, to confirm major reservoirs and the specific transmission routes; (4) Developing vaccines targeting dominant regional serovars/clades, such as *S*. Typhimurium ST313 in Africa and *S*. Enteritidis in China, optimized by targeting conserved immunological parameters.

However, the implementation challenge from genomic discovery to public health interventions remains, particularly in resource‐limited regions. Amplified by the dual threats of increasing human transmission and accelerating AMR, the crisis creates an urgent need to deploy cutting‐edge technologies. Innovations such as AI‐augmented surveillance, messenger RNA vaccine platforms, CRISPR‐based diagnostics, and phage therapy, supported by equitable global partnerships, are essential. As a pathogen that straddles the zoonotic and human realms, iNTS serves as a sentinel for the pathogens of ongoing threats, demanding a proactive, technologically‐enabled, and globally coordinated response that is not merely an option, but an imperative for global health security.

## AUTHOR CONTRIBUTIONS

MY, HW, LT, together wrote the initial draft, HZ and PX reviewed the manuscript.

## CONFLICT OF INTEREST STATEMENT

The authors declare no conflict of interest.

## FUNDING INFORMATION

This work was supported by the Chinese National Science Foundation (32573359), National Program on Key Research Project of China (grant no. 2022YFC2604201) and theEU's Horizon 2020 Research and Innovation Program under Grant Agreement No. 861917 ‐ SAFFI, Zhejiang Provincial Natural Science Foundation of China (grant no. LZ24C180002), and the Research Funds of Hangzhou Institute for Advanced Study, UCAS. The funders had no role in study design, data collection and analysis, decision to publish or preparation of the manuscript.

## ETHICS STATEMENT

None to declare.
